# A missing link in the estuarine nitrogen cycle?: Coupled nitrification-denitrification mediated by suspended particulate matter

**DOI:** 10.1038/s41598-018-20688-4

**Published:** 2018-02-02

**Authors:** Weijing Zhu, Cheng Wang, Jaclyn Hill, Yangyang He, Bangyi Tao, Zhihua Mao, Weixiang Wu

**Affiliations:** 1 Zhejiang Province Key Laboratory for Water Pollution Control and Environmental Safety Technology, Institute of Environmental Science and Technology, Zhejiang University, 866 Yuhangtang Road, Hangzhou, 310058 China; 2 Department of Zoology & Entomology, Rhodes University, PO Box 94, Grahamstown, 6140 South Africa; 3 State Key Laboratory of Satellite Ocean Environment Dynamics, Second Institute of Oceanography, State Oceanic Administration, 36 Baochu North Road, Hangzhou, 310012 China

## Abstract

In estuarine and coastal ecosystems, the majority of previous studies have considered coupled nitrification-denitrification (CND) processes to be exclusively sediment based, with little focus on suspended particulate matter (SPM) in the water column. Here, we present evidence of CND processes in the water column of Hangzhou Bay, one of the largest macrotidal embayments in the world. Spearman’s correlation analysis showed that SPM was negatively correlated with nitrate (*rho* = −0.372, *P* = 0.018) and marker genes for nitrification and denitrification in the water column were detected by quantitative PCR analysis. The results showed that *amoA* and *nir* gene abundances strongly correlated with SPM (all *P* < 0.01) and the ratio of *amoA*/*nir* strongly correlated with nitrate (*rho* = −0.454, *P* = 0.003). Furthermore, aggregates consisting of nitrifiers and denitrifiers on SPM were also detected by fluorescence *in situ* hybridization. Illumina MiSeq sequencing further showed that ammonia oxidizers mainly belonged to the genus *Nitrosomonas*, while the potential denitrifying genera *Bradyrhizobium*, *Comamonas*, *Thauera*, *Stenotrophomonas*, *Acinetobacter*, *Anaeromyxobacter*, *Sulfurimonas*, *Paenibacillus* and *Sphingobacterium* showed significant correlations with SPM (all *P* < 0.01). This study suggests that SPM may provide a niche for CND processes to occur, which has largely been missing from our understanding of nitrogen cycling in estuarine waters.

## Introduction

The amount of reactive nitrogen (N) ultimately ending up in estuarine and coastal ecosystems, which stems from human activity has increased extensively (over 150%) in the 20^th^ century. Excess N has resulted in increased eutrophication across numerous estuarine ecosystems and has consequently become a global matter of concern over the last several decades^1^. Estuarine eutrophication has resulted in a suite of environmental problems including oxygen depletion, algae blooms, loss of biodiversity, global acidification and even the establishment of invasive species^2,3^. Recently, the Bulletin of China’s Marine Environmental Status (2014) showed that anthropogenic pollution is of particular concern in China, with nearly 80% of Chinese estuaries subject to some level of eutrophication. Thus, understanding N transformation, as well as removal mechanisms in estuarine systems are critical for the maintenance and conservation of estuarine ecosystem health.

N biogeochemistry, which is almost exclusively reliant on reduction-oxidation (redox) reactions, is facilitated primarily by microorganisms^4^. Denitrification, the sequential reduction of nitrate (NO_3_^−^) to dinitrogen gas (N_2_) via oxidized intermediates, is considered to be the dominant loss pathway for fixed N in shallow coastal and estuarine systems^5^, although anammox (the anaerobic oxidation of ammonium) has been recently identified as an alternative microbial pathway of N_2_ production^6^. Two nitrite reductases—copper-containing and cytochrome *cd*_1_ nitrite reductases (*nirK* and *nirS*) are key enzymes in the denitrification pathway^7^. Nitrification, the two-step conversion of ammonium (NH_4_^+^) to NO_3_^−^ via nitrite (NO_2_^−^), is generally thought to play a critical role in the N cycle. Ammonia oxidation is considered to be the rate-limiting step of nitrification and is catalyzed by ammonia monooxygenase (AMO), which is encoded by the *amoA* gene from both archaea (AOA *amoA*) and bacteria (AOB *amoA*)^8^. When coupled with denitrification, nitrification partially mitigates the adverse effects of eutrophication, by removing bioavailable N from the water and releasing it to the atmosphere as N_2_O or N_2_.

In general, nitrification is an aerobic process and denitrification is an anaerobic one, and the two processes are often carried out in different ecological niches. Sediment, with substantial niche diversity (characterized by complex structural properties and sharp redox gradients), has been identified as the dominant sink for fixed N^9^. Coupled nitrification-denitrification (CND) processes in estuarine sediments contribute substantially to N loss, in some cases removing 10-80% of anthropogenic N pollution^10^. To date, there are a number of studies which have shown the importance of sediment-based CND in estuaries^11,12^; however there may be additional sites with comparable niche diversity in estuaries that may facilitate CND and subsequent N removal.

Strong tidal currents often resuspend fine particulates from the benthos, and the resulting suspended particulate matter (SPM) is a universal component of estuarine waters, particularly in macrotidal estuaries^13^. Composed of nutrients, organic micro-pollutants and heavy metals, SPM can affect material exchange and biogeochemical processes in estuarine systems^14^. In the maximum turbidity zone (MTZ), where primary production tends to be light-limited, the accumulating SPM is a substrate for most bacterial activity^15^. While a number of studies have followed the effects of particle-attached heterotrophic bacteria on estuarine carbon circulation^16,17^, the fate of excess N, driven by microbially-mediated processes related to SPM in estuarine environments, remains unclear. Does SPM provide adequate conditions for CND processes to occur in estuarine waters?

**Figure 1:**
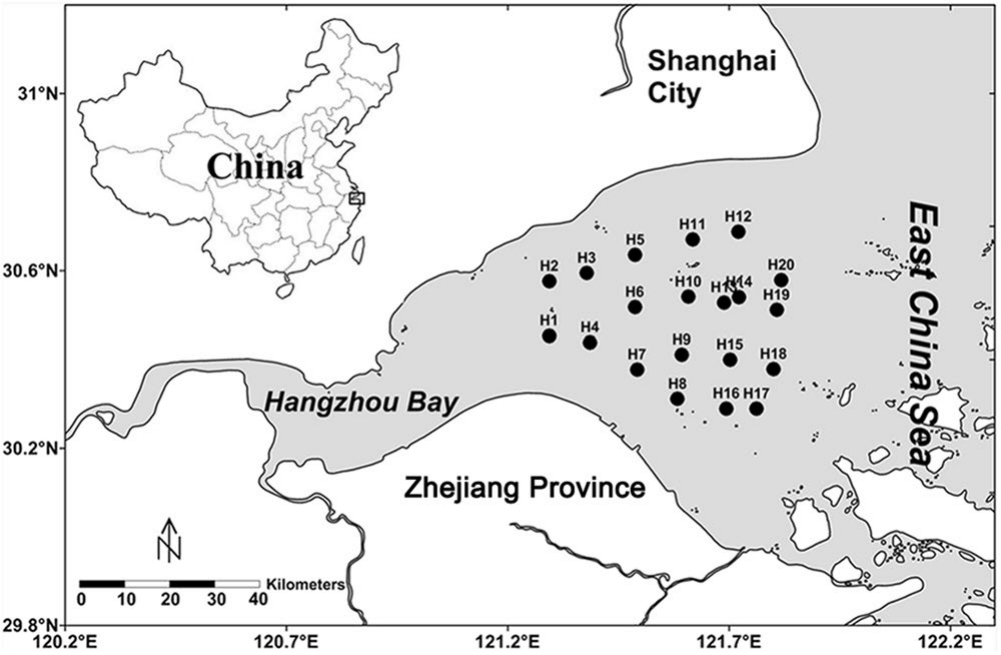
Map of Hangzhou Bay, China showing sampling sites on the west coast of the East China Sea. The Surfer 11 software (http://www.goldensoftware.com/products/surfer) was used to generate the map.

Hangzhou Bay is located in the northern Zhejiang Province of China and is adjacent to the East China Sea (Fig. 1). Covering an area of approx. 8,500 km^2^, it is one of the world’s largest macrotidal embayments. The tidal amplitude at the mouth is 3–4 m, and it exceeds 4–6 m further upstream. Tidal currents are mainly rectilinear and the maximal flood velocity exceeds 4.0 m/s^18^. Influenced by tidal currents and waves, Hangzhou Bay has a high carrying capacity for SPM, which is carried downstream from the Changjiang River and the Qiantang River^19^. The mean annual water discharge and sediment transport in Hangzhou Bay are 2.91 × 10^10^ m^3^ and 6.68 × 10^6^ tons, respectively^20^. The purpose of this study is to investigate the potential for CND processes mediated by SPM in the water column of Hangzhou Bay and provide new insight into the role of SPM in estuarine N cycle.

## Results

### Environmental parameters and correlation analyses

Environmental parameters in the water column of Hangzhou Bay are shown in Supplementary Table S1, across a scale of ~55 km. Ranging from 0.02 to 8.07 g/L, SPM of the surface water was significantly lower than that of the bottom water (Table 1; *F*_(1,39)_ = 26.869, *P* = 0.000) and the opposite result was seen for particulate organic carbon (POC) (Table 1; *F*_(1,39)_ = 40.301, *P* = 0.000). NO_3_^−^ accounted for 95.85–99.80% of the dissolved inorganic nitrogen (DIN) in the water column. However, NO_3_^−^ of the surface water was significantly higher than that of the bottom water (Table 1; *F*_(1,39)_ = 6.110, *P* = 0.018). The remaining environmental parameters, including temperature, salinity, dissolved oxygen (DO), pH, chlorophyll a (Chl a), NH_4_^+^ and NO_2_^−^, showed no significant differences between the surface and bottom water of Hangzhou Bay (Table 1).

**Table 1:** Means (±SD) of environmental parameters for samples taken from the surface and bottom waters and results of the one-way ANOVA (*F*) showing significant differences in environmental parameters with depth.

	Depth	Temperature	Salinity	DO	pH	Chl a	SPM	POC	NH_4_^+^	NO_2_^−^	NO_3_^−^
	(m)	(°C)	(ppt)	(mg/L)		(μg/L)	(g/L)	(g/g)	(μM)	(μM)	(μM)
mean ± SD^a^	0.05 ± 0.00	20.27 ± 1.47	21.77 ± 5.08	6.21 ± 0.70	8.01 ± 0.02	0.65 ± 0.32	0.36 ± 0.25	0.14 ± 0.03	0.92 ± 0.6	0.12 ± 0.10	119.07 ± 16.22
mean ± SD^b^	9.80 ± 2.24	19.57 ± 0.93	22.51 ± 4.69	6.16 ± 0.78	8.00 ± 0.02	0.60 ± 0.2	2.64 ± 1.95	0.09 ± 0.02	1.03 ± 1.02	0.16 ± 0.10	98.38 ± 33.73
*F*	**345.233** ^**^	3.237	0.230	0.053	2.967	0.323	**26.869** ^**^	**40.301** ^**^	0.186	1.455	**6.110** ^*^
*rho*	**0.760** ^**^	**−0.436** ^**^	0.152	−0.029	−0.209	−0.161	—	**−0.706** ^**^	0.027	**0.345** ^*^	**−0.372** ^*^

Spearman’s correlation coefficients (*rho*) between SPM concentration and other environmental parameters of the entire water column (pooled surface + bottom) are also presented.

^a^Environmental parameters of surface water column.

^b^Environmental parameters of bottom water column.

Data in bold indicate significant correlations, *P < 0.05; **P < 0.01.

Spearman’s correlation analyses between SPM and other environmental parameters (pooled surface and bottom) were performed (Table 1; *N* = 40). Strong positive correlation was identified between SPM and depth (Table 1; *rho* = 0.760, *P* = 0.000) and strong negative correlations were identified between SPM and both temperature and POC (Table 1; *rho* = −0.436, *P* = 0.005 and *rho* = −0.706, *P* = 0.000). Furthermore, NO_2_^−^ and NO_3_^−^ showed weak correlations with SPM (Table 1; *rho* = 0.345, *P* = 0.029 and *rho* = −0.372, *P* = 0.018), while no relationship between SPM and NH_4_^+^ was observed. It is worth noting that no correlation was found between SPM and NO_3_^−^ in surface water (*N* = 20) or bottom water (*N* = 20) when compared alone (data not shown).

### Abundances of nitrifying and denitrifying genes and correlation analyses

**Figure 2:**
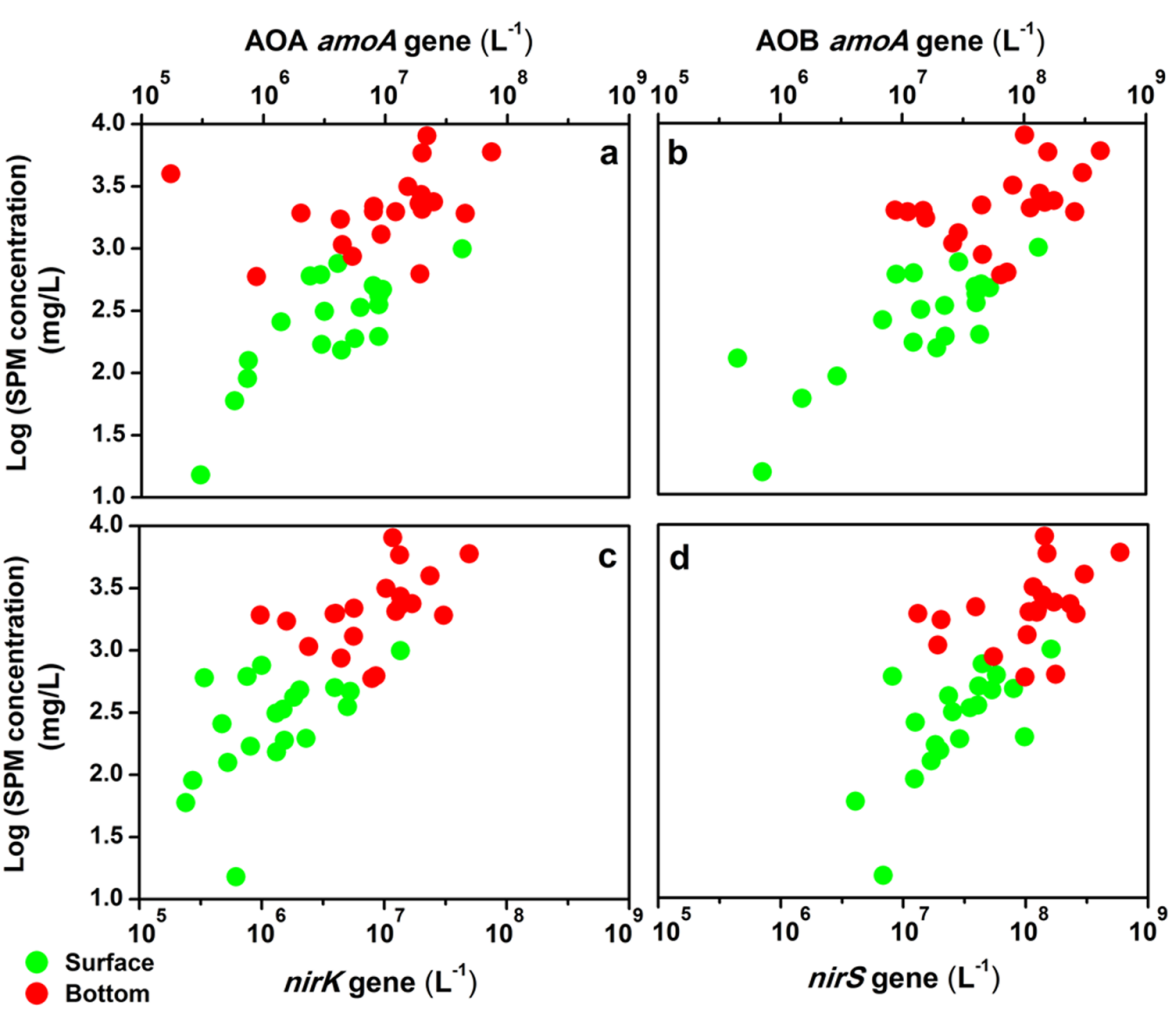
The abundances and distributions of (**a**) AOA *amoA*, (**b**) AOB *amoA*, (**c**) *nirK* and (**d**) *nirS* genes along SPM concentration gradient in Hangzhou Bay surface (*N* = 20) and bottom (*N* = 20) water column.

Quantitative PCR (qPCR) analysis was used to estimate the abundances of the key nitrifying (AOA *amoA* and AOB *amoA*) and denitrifying (*nirK* and *nirS*) genes in the water samples obtained from the study area. Among the nitrifying genes, at each site, the abundance of AOB *amoA* gene (4.46 × 10^5^−4.22 × 10^8^ copies/L) was higher than that of AOA *amoA* gene (1.74 × 10^5^−7.43 × 10^7^ copies/L), with the exception of sample H4_S (Supplementary Table S2). Among the denitrifying genes, *nirS* gene appeared to be more numerous, with copy numbers ranging from 4.07 × 10^6^ to 5.90 × 10^8^ per liter of water, while copy numbers of *nirK* gene ranged from 2.39 × 10^5^ to 4.95 × 10^7^ per liter of water (Supplementary Table S2). The relative *nirS*/*nirK* ratios ranged from 6.90 to 76.29 in all samples, showing the clear dominance of *nirS* gene in the water column of Hangzhou Bay. Moreover, along the SPM gradient, both nitrifying and denitrifying genes showed significantly higher abundance of the bottom water than that of the surface water (Fig. 2). In addition, the abundance of anammox bacterial 16 S rRNA gene, ranging from 3.29 × 10^4^ to 9.81 × 10^6^ copies/L, was 1~2 orders of magnitude lower than the abundances of *nir* genes (Supplementary Table S2).

In order to explore the relationships between gene abundances and environmental parameters, Spearman’s correlation analyses were performed (Table 2; *N* = 40). The abundances of all functional genes showed strong positive correlations with SPM and depth (Table 2; all *P* < 0.01). Furthermore, negative correlations between NO_3_^−^ and AOB *amoA* and *nirK* gene abundances (Table 2; *rho* = −0.320, *P* = 0.044 and *rho* = −0.323, *P* = 0.042) were observed; however, no significant correlation was found between NO_3_^−^ and AOA *amoA* or *nirS* gene abundance (Table 2). Temperature and POC were also found to be negatively correlated with the abundances of all functional genes (Table 2; *P* < 0.05 or *P* < 0.01). Analyses of the gene copy ratios showed that the ratios of AOB *amoA*/AOA *amoA* and *nirK*/*nirS* were positively correlated with SPM (Table 2; *rho* = 0.495, *P* = 0.001 and *rho* = 0.403, *P* = 0.010), and negatively correlated with POC and NO_3_^−^ (Table 2; *P* < 0.05 or *P* < 0.01). In addition, *amoA*/*nir* value showed strong negative correlation with NO_3_^−^ (Table 2; *rho* = −0.454, *P* = 0.003).

**Table 2:** Spearman’s correlation coefficients (*rho*) between environmental parameters and gene abundances and the ratios of target genes across sampling sites (pooled surface + bottom samples).

	Depth	Temperature	Salinity	DO	pH	Chl a	SPM	POC	NH_4_^+^	NO_2_^−^	NO_3_^−^
	(m)	(°C)	(ppt)	(mg/L)		(μg/L)	(g/L)	(g/g)	(μM)	(μM)	(μM)
Abundance											
AOA *amoA*	**0.397** ^**^	**−0.397** ^*^	0.180	−0.033	−0.012	−0.060	**0.601** ^**^	**−0.390** ^**^	0.056	0.203	−0.084
AOB *amoA*	**0.562** ^**^	**−0.526** ^**^	0.268	−0.111	0.105	−0.230	**0.697** ^**^	**−0.467** ^**^	−0.087	0.190	**−0.320** ^*^
*nirK*	**0.644** ^**^	**−0.416** ^**^	0.153	−0.171	0.007	−0.127	**0.775** ^**^	**−0.586** ^**^	−0.150	0.175	**−0.323** ^*^
*nirS*	**0.548** ^**^	**−0.395** ^*^	0.128	−0.088	−0.073	−0.132	**0.746** ^**^	**−0.546** ^**^	−0.136	0.127	−0.178
Ratio											
AOB *amoA*/AOA *amoA*	**0.476** ^**^	**−0.414** ^**^	0.286	0.026	0.133	−0.226	**0.495** ^**^	**−0.342** ^*^	0.056	0.073	**−0.339** ^*^
*nirK*/*nirS*	**0.459** ^**^	−0.269	0.192	−0.304	0.233	−0.122	**0.403** ^**^	**−0.316** ^*^	−0.145	−0.022	**−0.427** ^**^
*amoA*^a^/*nir*^b^	0.153	**−0.491** ^**^	**0.345** ^*^	−0.252	0.280	−0.274	0.249	−0.118	0.196	0.176	**−0.454** ^**^

^a^Represents the sum of AOB *amoA* and AOA *amoA* gene abundances.

^b^Represents the sum of *nirK* and *nirS* gene abundances.

Data in bold indicate significant correlations, ^*^P < 0.05, ^**^P < 0.01.

### Bacterial community structures and RDA analysis

The Illumina MiSeq platform produced ~1,500,000 raw reads of the V4 amplicons. After removing the short and low-quality reads, 34,117–69,053 bacterial reads of each sample were available for further analyses. The numbers of OTUs, Chao 1 and Shannon’s indices at cutoff levels of 3% are summarized in Supplementary Table S3. On the basis of OTU numbers, the H7_B sample had the richest abundance, and declined to less than half that in H19_S sample. The comparison between surface and bottom water samples showed that the latter contained more bacterial richness than the former at most of the sampling sites (Supplementary Table S3). Very similar trends in the Chao 1 index were observed in comparison with OTU richness (Supplementary Table S3). Shannon diversity indices also indicated a higher bacterial diversity for the bottom water samples relative to the surface at all the sampling sites (Supplementary Table S3).

**Figure 3:**
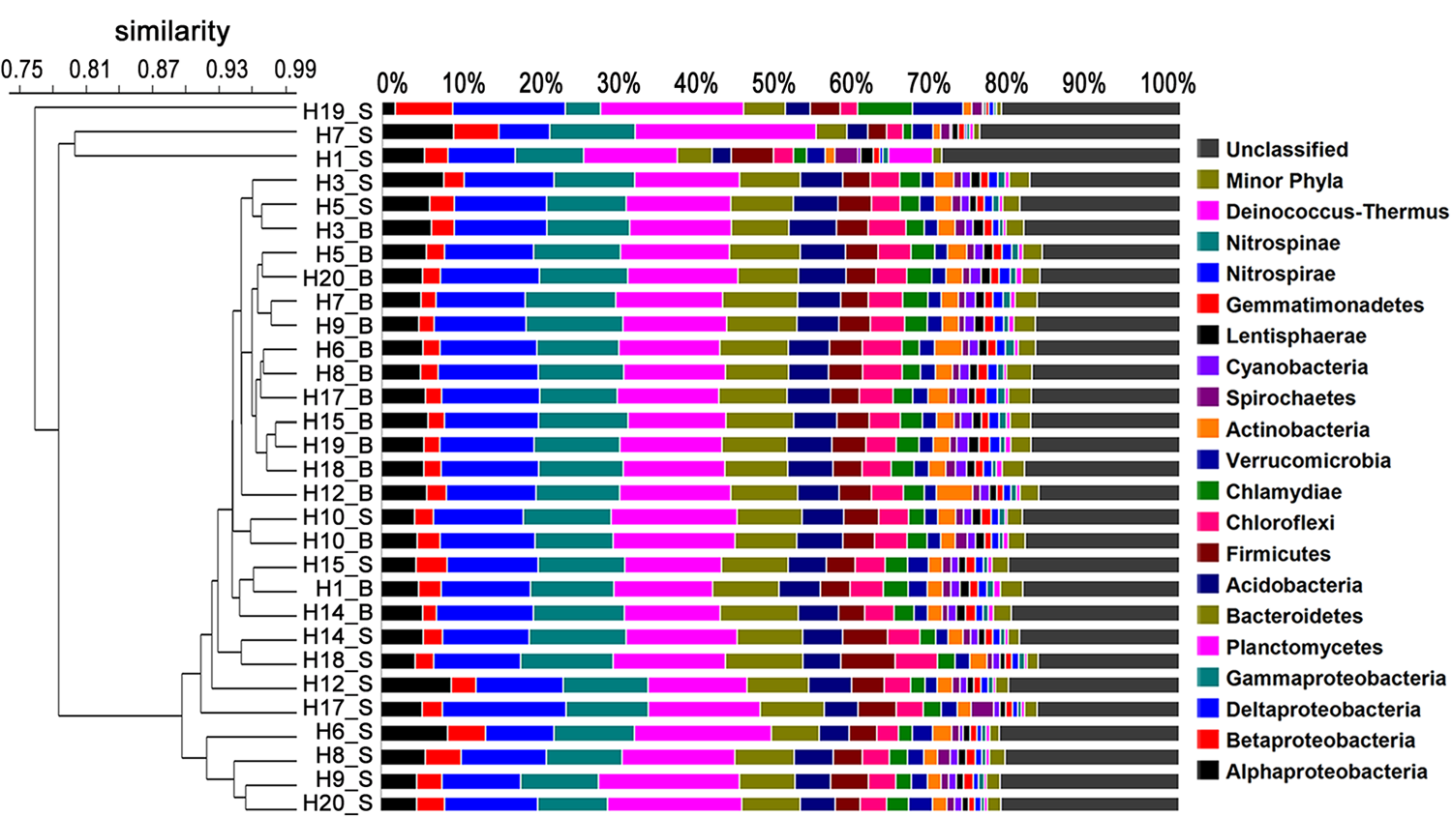
Dendrogram of hierarchical clustering of bacterial community structure based on Bray-Curtis similarity. Bacterial taxonomic information is shown at the phylum level (and subdivision level for Proteobacteria). Taxa represented occurred at >1% abundance in at least one sample. Minor phyla refer to the taxa with their maximum abundance <1% in any sample.

Cluster analysis at the phylum level revealed similarities of bacterial community structure in each sample of Hangzhou Bay (Fig. 3). In contrast to the surface water layer, bacterial communities in the bottom water layer were relatively stable and showed little variability, with almost all samples clustering together (Fig. 3). Proteobacteria was the most abundant phylum across all water samples, accounting for 28.56–35.32% of the total effective bacterial sequences. Within the Proteobacteria group, Deltaproteobacteria (6.37–15.52%, averaging 11.36%) was the most dominant class, followed by Gammaproteobacteria (4.44–12.19%, averaging 10.34%), Alphaproteobacteria (1.61–8.94%, averaging 5.41%) and Betaproteobacteria (1.73–7.19%, averaging 2.92%). The other dominant phyla were Planctomycetes (11.73–22.69%, averaging 14.10%), Bacteroidetes (3.86–9.82%, averaging 7.76%), followed by a few other major phyla (abundance >1% in each sample), including: Acidobacteria, Firmicutes, Chloroflexi, Chlamydiae and Verrucomicrobia. A few phyla (e.g. Actinobacteria, Spirochaetes, Cyanobacteria, Lentisphaerae, Gemmatimonadetes, Nitrospirae, Nitrospinae and Deinococcus-Thermus) were only major contributors (abundance >1%) to phyletic compositions of single samples.

**Figure 4:**
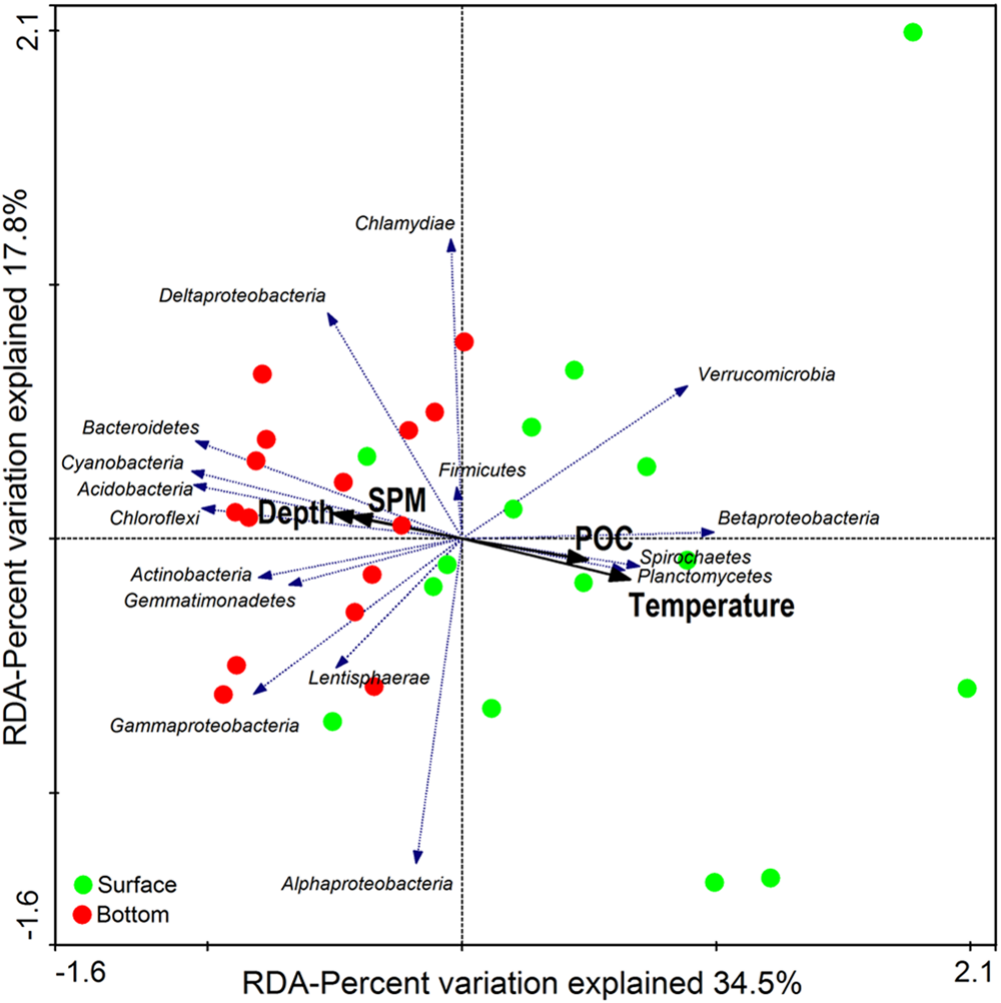
Redundancy analysis of the relationship between environmental parameters (black arrows) and bacterial community distributions of surface (green circles) and bottom (red circles) water column of Hangzhou Bay. Taxonomic information is shown at the phylum level (and subdivision level for Proteobacteria). Only *P* value of environmental parameter <0.05 (999 times Monte-Carlo permutation test) and average abundance of taxa >1% are shown.

Redundancy analysis (RDA) was conducted to identify the potential effect of environmental parameters on bacterial community patterns. Overall, the first two RDA axes explained 52.3% of the total variation, and RDA1 axis clearly distinguished the bacterial distributions of surface water from that of bottom water. Of all the environmental parameters measured, depth (*F* = 5.492, *P* = 0.001), temperature (*F* = 5.150, *P* = 0.005), SPM (*F* = 3.836, *P* = 0.033) and POC (*F* = 10.413, *P* = 0.001) appeared to be the major drivers influencing bacterial distributions, while other variables (e.g., salinity, DO, pH, Chl a, NH_4_^+^, NO_2_^−^ and NO_3_^−^) had weaker effects on bacterial assemblages (Fig. 4).

### Sequences belong to nitrifying and potential denitrifying genera

Taxonomically, AOB are phylogenetically restricted to two lineages within the Proteobacteria: the Betaproteobacteria, including the genera *Nitrosomonas*, *Nitrosospira*, *Nitrosovibrio* and *Nitrosolobus*^21^; and the Gammaproteobacteria, including the genus *Nitrosococcus*^22^. The Betaproteobacterial *Nitrosomonas* and *Nitrosospira*, and the Gammaproteobacterial *Nitrosococcus* were observed in this study. The *Nitrosomonas* and *Nitrosococcus* groups were the two dominant genera of AOB, accounting for 0.06–5.25% and 0.03–0.17% of the total sample sequences respectively, while the genus *Nitrosospira* was nearly absent in this study (Supplementary Figure S1a). In addition, the relative abundance of *Nitrosomonas* showed strong negative correlations with SPM, NO_2_^−^ and AOB *amoA* gene (Supplementary Table S4; all *P* < 0.01), and a weak positive correlation with NO_3_^−^ (Supplementary Table S4; *rho* = 0.442, *P* = 0.014). While, the relative abundance of *Nitrosococcus* was only positively correlated with SPM (Supplementary Table S4; *rho* = 0.422, *P* = 0.020).

Previous studies provided a list of ~100 denitrifying bacterial genera^23–26^, among which 44 genera were detectable in the water column of Hangzhou Bay (Supplementary Figure S1b). In this study, the majority of the potential denitrifying genera belonged to Proteobacteria (31 genera; 0.47–1.61% of the total 16 S rRNA sequences). The second largest group of denitrifiers was categorized as Bacteroidetes (4 genera; 0.17–0.83%), followed by the group of Firmicutes (5 genera; 0.08–0.46%) and Actinobacteria (4 genera; 0–0.05%) (Supplementary Figure S1b). Among the 44 denitrifying genera, many of them showed strong positive correlations with SPM (*P* < 0.01), which mainly included (i.e. average abundance >0.05%) the genera *Bradyrhizobium* of Alphaproteobacteria; *Comamonas* and *Thauera* of Betaproteobacteria; *Stenotrophomonas* and *Acinetobacter* of Gammaproteobacteria; *Anaeromyxobacter* of Deltaproteobacteria; *Sulfurimonas* of Epsilonproteobacteria; *Paenibacillus* of Firmicutes; and *Sphingobacterium of* Bacteroidetes (Supplementary Table S5).

### *In situ* characterization of nitrifying and denitrifying bacteria

**Figure 5:**
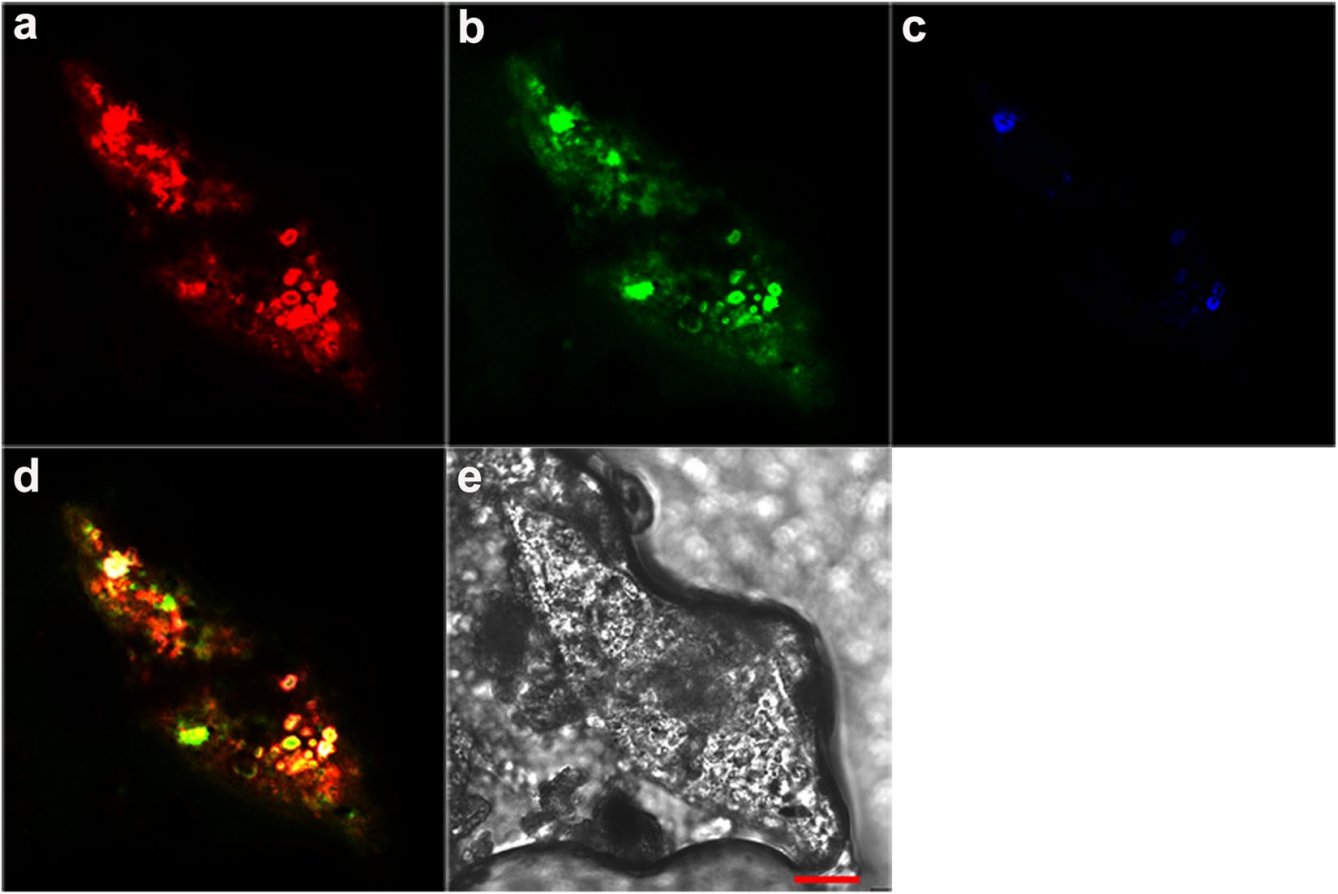
Simultaneous *in situ* hybridization of SPM samples in the water column of Hangzhou Bay. Fluorescence micrograph of (**a**) ammonia-oxidizing bacteria hybridization with Cy3-labeled probe NSO190 (red); (**b**) acetate-denitrifying cluster hybridization with FAM-labeled probe DEN124 (green); (**c**) methanol-denitrifying cluster hybridization with Cy5-labeled probe DEN67 (blue); (**d**) combined image of the three fluorescence micrographs, where the yellow cell aggregates are double labeled with NSO190 and DEN124, and the white cell aggregates are tripled labeled with NSO190, DEN124 and DEN67. A phase contrast-micrograph of the floc section, where the red bar = 20 μm, is depicted in (**e**).

The presence of nitrifiers and denitrifiers in the bacterial consortia of the SPM was examined by fluorescence *in situ* hybridization (FISH) analysis. *In situ* hybridization clearly indicated that AOB and denitrifiers were not uniformly distributed within the SPM, and AOB were in greater abundance than acetate/methanol-denitrifying cells (Fig. 5a–c and Supplementary Figures S2a–c). Specifically, the majority of NSO190 probe-stained AOB and DEN124 probe-stained acetate-denitrifying cells formed irregularly shaped (2–10 μm), dense aggregates, with ammonia-oxidizing microcolonies found mainly in the outer part of the aggregate and acetate-denitrifying cells in the middle (Fig. 5d and Supplementary Figure S2d). Though fewer cells hybridizing with probe DEN67 were detected (Fig. 5c and Supplementary Figure S2c), the white signals in the figures indicated that the methanol-denitrifying cells also existed in inner sections of the aggregates.

## Discussion

Although a previous study has shown that NH_4_^+^ is the primary anthropogenic nutrient entering Hangzhou Bay from the Qiantang River^27^, NO_3_^−^ was found to be the most important form of DIN in the water column of Hangzhou Bay. Chl a concentrations (0.26–1.75 μg/L) in the water column were much lower than those measured in other estuaries^28,29^, and is likely indicative of poor light availability which severely limits phytoplanktonic uptake of NH_4_^+ ^^30^. Furthermore, no negative relationship between Chl a and NH_4_^+^ or NO_3_^−^ was found (data not shown). It is likely that microbial nitrification is a key process for the fate of NH_4_^+^ in the water column. SPM was found to be negatively correlated with NO_3_^−^ and positively correlated with NO_2_^−^, potentially linking SPM-attached denitrifiers to nitrate reduction. We investigated these possibilities by quantifying the abundances of nitrifiers and denitrifiers in the water column and tested their relationships with the measured environmental parameters. High abundances of *amoA* and *nir* genes were detected in both the surface and bottom layers of the water column, with the abundances of AOB *amoA* and *nirK* genes and the ratio of *amoA*/*nir* significantly correlating with NO_3_^−^, showing links between nitrifiers/denitrifiers and available N^31^. Although correlations between SPM and indicators of denitrifying bacteria do not necessarily indicate causation, these results provide insight on the potential possibility of SPM to act as an important site for CND processes in the water column of Hangzhou Bay.

Crucial environmental parameters known to influence abundance and diversity of nitrifiers and denitrifiers mainly include temperature, salinity, oxygen and nutrient availability^5,32^. Nevertheless, in this study, the abundances of nitrifying and denitrifying genes were strongly correlated with SPM concentration. Moreover, the results of the RDA suggested that SPM played an important role in shaping the bacterial communities in the water column. In particular, the phyla (or class) *Bacteroidetes*, *Planctomycetes* and *Deltaproteobacteria* were the dominant community members and have recently been shown to be associated with larger particle size fractions^33^. Overall, these results suggest that SPM may provide a large area for nitrifiers and denitrifiers to co-exist, shaping nitrifying and denitrifying communities in estuarine water. Meanwhile, aggregates consisting of outer nitrifiers and inner denitrifiers detected by FISH analysis indicate that anoxic/low oxygen microsites probably exist inside the SPM. According to Jia *et al*. (2016), high O_2_ influx around suspended particles and decreasing DO concentration suggested that oxygen was being consumed near the particle’s surface by nitrification and/or microbial respiration^34^. They reported that particles with diameters <20 μm had the largest overall increase in O_2_ influx. Schramm *et al*. had also reported that the volumetric respiration rate was negatively correlated with floc size^35^. Accordingly, SPM in Hangzhou Bay is predominantly composed of fine and medium silt, and the grain size of over 90% of the SPM is less than 20 μm^18,36^. FISH analyses in the present study support this, showing that the size of most aggregates on SPM were far less than 20 μm (Fig. 5 and Supplementary Figure S2). Based on these results, we suggest that SPM could not only provide large areas for the co-existence of nitrifiers and denitrifiers, but may also provide redox conditions for CND processes to occur in oxic water.

Numerous earlier studies suggest that AOA phylotypes are more abundant than AOB in estuarine ecosystems^37,38^. However, our qPCR estimates indicated that the abundance of bacterial *amoA* gene was higher than that of archaeal *amoA* gene in all samples (with a single exception). Moreover, the abundance of AOB *amoA* gene significantly correlated with NO_3_^−^, while the abundance of AOA *amoA* gene did not. Thus, it is possible that nitrification may be driven by bacteria rather than archaea in the water column of Hangzhou Bay. Furthermore, the abundance of *Nitrosomonas* was positively correlated with NO_3_^−^ and negatively correlated with NO_2_^−^, suggesting it may be the predominant AOB during nitrification process. Similar to previous studies, members of the *Nitrosomonas* group appear to be dominant in most terrestrial and aquatic environments^39^. Despite *Nitrosomonas* being the most dominant AOB in the water column, the genus *Nitrosococcus* was dominant in the sediment (Supplementary Figure S3). This may partly explain why the abundance of *Nitrosomonas* was negatively correlated with SPM.

Since the concentration of Chl a in the water column was very low, it seemed unlikely that nitrate was assimilated by algae. Additionally, the abundance of *nirK* gene was negatively correlated with NO_3_^−^, while the *nirS* gene was not. Thus, it is possible that the decrease of nitrate concentration in the system is mainly caused by *nirK*-type denitrifiers on SPM. Though *nirS* gene appears to be more abundant in nature^40^, the activity of cytochrome *cd*_1_ nitrite reductases (NirS) was more likely to be repressed than that of copper-containing nitrite reductases (NirK) in the oxic water of Hangzhou Bay. As Körner and Zumft (1989)^41^ pointed out, the threshold values for synthesis of copper-containing nitrite reductases (NirK) was 5 mg of O_2_ per liter, and several studies from different environments have also shown that bacteria carrying the *nirK* gene were not particularly sensitive to oxygen^42,43^. The higher abundance of *nirS* gene in the water column of Hangzhou Bay was probably a result of sediment resuspension, as the abundance of *nirS*-type denitrifiers (1.90 × 10^6^–4.69 × 10^7^ copies/g dry sediment) was much higher than that of *nirK*-type denitrifiers (1.21 × 10^4^−3.27 × 10^6^ copies/g dry sediment) in the sediment of Hangzhou Bay (Supplementary Figure S4). While not definitive, many of the denitrifying genera identified in this study showed strong positive correlations with SPM, and are potentially indicative of denitrification process, providing a new direction for inquiry into CND processes in aerobic water columns.

Recently, a series of ^15^N isotopic tracer studies have suggested that the nitrification/denitrification rates in oxic water are significantly affected by the abundances of nitrifiers/denitrifiers on the suspended particles^44–47^. Although the current study did not make direct measurement of nitrification or denitrification rate, on the basis of results above, we assume that the abundances of nitrifiers/denitrifiers on SPM can also reflect (to some extent) nitrification/denitrification rates in the turbid water of Hangzhou Bay. The microbial mechanisms behind CND processes are potentially as follows: in the case of water column with high oxygen concentration, SPM can provide large areas for the co-existence of nitrifying and denitrifying bacteria. As is known to all, NO_2_^−^ is difficult to accumulate in the oxic environment and will finally be oxidized to NO_3_^−^. With the number of AOB being far greater than that of *nirK*-type denitrifiers, it is not NO_2_^−^ but NO_3_^−^ that accumulated in the system, accounting for 95.85–99.80% of the total inorganic nitrogen. When SPM increased, more low/anoxic microsites could be generated, caused by respiration of heterotrophic bacteria and transport limitations^34^. Although overall, the number of AOB significantly increased, the abundance of *Nitrosomonas* (the dominant AOB) itself, significantly decreased, leading to the decrease in NO_3_^−^ production. Meanwhile, the number of *nirK*-type denitrifiers significantly increased, and the increase of both low/anoxic microsites and denitrifiers within the SPM promotes denitrification, leading to the increase in NO_3_^−^ removal. In combination, both processes eventually resulted in a significant decrease in NO_3_^−^ (Supplementary Figure S5). Correspondingly, the decrease of POC concentration suggested an increasing consumption of POC by denitrification and/or heterotrophic bacterial respiration.

With the caveat that correlations between SPM and indicators of denitrifying bacteria do not necessarily indicate causation, the current study reports for the first time that SPM may provide the conditions for CND processes to occur in the water column of Hangzhou Bay. These preliminary insights on the coupling of nitrification and denitrification processes mediated by SPM, help to resolve our understanding of N cycle in estuarine ecosystems. High SPM concentration, caused by erosion and bottom sediment resuspension, could accelerate N transformation and the subsequent removal processes in turbid estuaries and should be included in budgets of riverine N flux to coastal oceans. The findings shed further light on our conceptual views of N fluxes and transformation in estuarine ecosystems and provide another avenue of consideration with respect to CND processes. However, the paucity of direct measurements of CND rates of SPM hinders further interpretations of this current dataset. Quantifying the relationship between direct measurements of nitrification/denitrification rates of SPM and the associated microorganisms, through the application of ^15^N isotopic tracer technologies for example, may help to elucidate how these coupling processes respond to SPM variability in the turbid estuarine waters.

## Materials and Methods

### Sampling

Samples of surface (at 0.5 m depth) and bottom water (1 m above the benthos) were collected from twenty sites along Hangzhou Bay during a 7 day cruise in May 2014, when the phytoplankton quantity was quite low^48^ (Fig. 1). All water samples were collected with a 5-L Niskin bottle (Tianjin test center, Tianjin, China), and then sampled from the Niskin bottle with a plastic syringe. Standard oceanographic properties including water temperature, salinity, DO and pH were measured immediately using a Horiba U-52 water quality checker (Horiba, Kyoto, Japan). The concentrations of Chl a (µg/L), SPM (g/L), POC (g per g SPM) and nutrients (NH_4_^+^, NO_2_^−^ and NO_3_^−^; µM) were measured following standard protocols^49^. Water samples for nucleic acids analysis and SPM samples for FISH analysis were collected onto 0.2-μm and 0.45-μm polycarbonate GTTP membranes (Millipore, Billerica, MA, USA) respectively and preserved at −80 °C.

### DNA extraction

Total nucleic acids of the water samples were extracted directly from the 0.2-μm membranes using a FastDNA spin kit for soil (Qbiogene, Carlsbad, CA, USA), following the manufacturer’s instructions. Duplicate DNA extractions for each water sample were performed. DNA quality was detected through 1% agarose gel electrophoresis which was stained with SYBR Safe DNA Gel Stain (Invitrogen, Carlsbad, CA, USA). The duplicate DNA extractions were then merged together, and stored at −80 °C for subsequent molecular analysis.

### QPCR analysis

To examine the spatial variation of community size for nitrifying and denitrifying groups in the water column of Hangzhou Bay, the abundances of AOA *amoA*, AOB *amoA*, *nirK* and *nirS* genes were quantified using qPCR. Serial tenfold dilutions (10^−1^ to 10^−5^) of linearized plasmids containing cloned AOA *amoA*, AOB *amoA*, *nirK* and *nirS* genes were used to obtain standard curves. All sample and standard reactions were performed in triplicate using CFX 96 C 1000^TM^ Thermal Cycler (Bio-Rad, Hercules, CA, USA), and average values were calculated. Each reaction was performed in 20 μL containing 2 μL of total DNA template, 0.4 μL of each primer (10 mM) and 10 μL of SYBR Premix Ex Taq (Takara, Tokyo, Japan). Primers used are listed in Supplementary Table S6. The PCR cycle started with 3 min at 95 °C, followed by a total of 40 cycles of 10 s at 95 °C, 30 s at 55 °C for AOB *amoA* gene (57 °C for AOA *amoA* and *nirS* genes, and 58 °C for *nirK* gene) and 30 s at 72 °C. The specificity of amplification was checked by the observation of melt curves. PCR amplification efficiencies were 83–100.7% with correlation coefficients (R^2^) over 0.99 for all calibration curves.

### Illumina MiSeq sequencing and sequence analysis

Bacterial communities were investigated at 15 sites for both surface and bottom layers of the water column of Hangzhou Bay, using high-throughput sequencing according to the protocols described by Caporaso *et al*.^50^. The V4 regions of bacterial 16 S rRNA gene were amplified from the DNA extracts using primers 520 F (5′-barcode-AYTGGGYDTAAAGNG-3′) and 802 R (5′-TACNVGGGTATCTAATCC-3′). The barcode is a seven-base sequence unique to each sample. A 25 μL reaction contained 1.25 U of Q5 polymerase (Stratagene, La Jolla, CA, USA), 5 μL Q5 reaction buffer (5×), 5 μL Q5 GC high Enhancer (5×), 0.2 mM of dNTPs (TaKaRa), 0.4 mM of each primer and 40 ng of total DNA. PCR amplification was conducted under the following conditions: initial denaturation at 98 °C for 5 min; 27 cycles at 98 °C for 30 s, 50 °C for 30 s and 72 °C for 30 s; and a final extension at 72 °C for 5 min. PCR amplicons were purified using AxyPrep DNA Gel Extraction Kits (Axygen, Union City, CA, USA) and quantified on a FLx800 Fluorescence Microplate Reader (BioTek, Winooski, VT, USA). Amplicons from different water samples were then pooled to achieve equal mass concentrations in the final mixture, which was sent out for high-throughput sequencing on the Illumina MiSeq platform (Personalbio, Shanghai, China). Sequences are available in the NCBI short-read archive database (Accession Number: SRP091596).

The sequencer generated 1,459,768 reads of 16 S rRNA gene from 30 samples. Raw sequences were de-multiplexed and quality-filtered using the default parameters in Qiime version 1.7.0^51^. Criteria used for the filtering step were recommended by Bokulich *et al*.^52^. The remaining PCR chimeras were removed using the uchime method in mothur version 1.31.2^53^. Bacterial sequences were then clustered into operational taxonomic units (OTUs; 97% similarity) with uclust in Qiime^54^. A bootstrap cutoff of 50% suggested by the Ribosomal Database Project (RDP) was applied for taxonomic assignment^55^. On the basis of OTU numbers, the alpha diversity measures (Chao 1 and Shannon index) were calculated in mothur^56^.

### FISH analysis

SPM samples were fixed on ice for 3 h in freshly prepared 4% paraformaldehyde solution and subsequently rinsed with phosphate-buffered saline. For *in situ* hybridization, 4 µL of each fixed sample was spotted onto adhesion microscope slides. Hybridization of the SPM samples was performed according to the procedure described by Amann (1995)^57^. Three 16 S rRNA-targeted oligonucleotide probes were used for *in situ* detection of nitrifying and denitrifying bacteria: (1) Cy3-labeled NSO190 probes: specific for ammonia-oxidizing β-subclass Proteobacteria (2) Cy5-labeled DEN67 probes: specific for methanol-denitrifying cluster and (3) FAM-labeled DEN124 probes: specific for acetate-denitrifying cluster. The specificities, sequences and hybridization conditions of all probes are shown in Supplementary Table S7. All hybridizations were performed at a temperature of 50 °C. Subsequently, fluorescent and phase-contrast images were recorded using a multiphoton confocal LSM 780 NLO microscope system (Carl Zeiss AG, Oberkochen, Germany).

### Data analysis

To assess the variations in environmental parameters between surface and bottom water layers, we used a one-way analysis of variance (ANOVA). Most of the variables were non-normal, except Chl a and NO_3_^−^. Spearman’s correlation analyses were used to test the correlations between SPM and other environmental parameters (depth, temperature, salinity, DO, pH, Chl a, POC, NH_4_^+^, NO_2_^−^ and NO_3_^−^); between gene abundances (or gene copy ratios) and environmental parameters (depth, temperature, salinity, DO, pH, Chl a, SPM, POC, NH_4_^+^, NO_2_^−^ and NO_3_^−^); between the abundance of nitrifying genera and SPM, NH_4_^+^, NO_2_^−^, NO_3_^−^ and AOB *amoA* gene abundance; and finally between the abundance of denitrifying genera and SPM. All calculations were performed using IBM SPSS statistics 20.0 software.

To compare the bacterial community in different samples, hierarchical clustering was performed in the program PAST^58^ based on Bray-Curtis similarity index and unweighted pair group method average (UPGMA) algorithm. To test the influences of environmental parameters (depth, temperature, salinity, DO, pH, Chl a, SPM, POC, NH_4_^+^, NO_2_^−^ and NO_3_^−^) on the distributions of bacterial phylotypes, redundancy analysis (RDA) was performed using the software Canoco 5.0^59^. To meet assumptions, all measured environmental parameters were standardized before analyses.

## Electronic supplementary material

supporting information
