# Supplementary material for: A missing link in the estuarine nitrogen cycle?: Coupled nitrification-denitrification mediated by suspended particulate matter
Source: Sci Rep. 2018 Feb 2;8:2282. doi: 10.1038/s41598-018-20688-4 (PMC5797115; doi:10.1038/s41598-018-20688-4)
Supplement: Supplementary file 1 — supporting information [file 41598_2018_20688_MOESM1_ESM.pdf]

Supporting Information

A missing link in the estuarine nitrogen cycle?: Coupled nitrification-denitrification mediated by suspended particulate matter

Weijing Zhu[1], Cheng Wang[1], Jaclyn Hill[2], Yangyang He[1], Bangyi Tao[3], Zhihua Mao[3*], Weixiang Wu[1*]

[1]*Zhejiang Province Key Laboratory for Water Pollution Control and Environmental Safety Technology, Institute of Environmental Science and Technology, Zhejiang University, 866 Yuhangtang Road, Hangzhou 310058, China*
[2]*Department of Zoology & Entomology, Rhodes University, PO Box 94, Grahamstown 6140, South Africa*
[3]*State Key Laboratory of Satellite Ocean Environment Dynamics, Second Institute of Oceanography, State Oceanic Administration, 36 Baochu North Road, Hangzhou 310012, China*

Corresponding Author
[*]Phone: +86 571 88982020; fax: +86 571 88982020; e-mail: weixiang@zju.edu.cn (W. Wu);
Co-corresponding Author
[*]Phone: +86 571 89163115; fax: +86 571 88071539; e-mail: mao@sio.org.cn (Z. Mao);

Quantitative PCR (qPCR)

The abundance of anammox bacteria (AMB 16S rRNA gene) in the water column was also quantified using qPCR. The primers used are listed in Supplementary Table S6.

Sediment collection, DNA extraction, Quantitative PCR and Illumina Miseq sequencing

Sediment cores were collected at nine of the twenty sites in Hangzhou Bay (sites H1, H6, H7, H8, H9, H10, H14, H15 and H16; Figure 1). The top layer of sediments were sliced and immediately put into sterile plastic bags. The sediment samples were then frozen at -20 °C for later DNA extractions.

DNA was extracted from each sediment sample using the FastDNA spin kit for soil (Qbiogene, Carlsbad, CA, USA), following the manufacturer's instructions. DNA extractions were subsequently quantified using a Nano-Drop spectrophotometer (Nanodrop, Wilmington, DE, USA). The sediment DNA extractions were then stored at -80 °C for subsequent molecular analysis.

The procedures of qPCR and Illumina Miseq sequencing analyses for sediment DNA samples were identical to those procedures for the water DNA samples described in the manuscript.

Supplementary Table S1 Coordinates and mean environmental parameters of the sampling sites in the water column of Hangzhou Bay

Sample ID	Latitude (ºN)	Longitude (ºE)	Depth (m)	Temperature (ºC)	Salinity (ppt)	DO (mg/L)	pH	Chl a (μg/L)	SPM (g/L)	POC (g/g)	NH_4^+ (μM)	NO_2^- (μM)	NO_3^- (μM)
H1_S	30.45	121.29	0.50	23.40	12.62	6.06	8.00	0.76	0.09	0.22	0.81	0.41	139.16
H2_S	30.58	121.29	0.50	21.40	17.76	6.49	8.01	0.65	0.31	0.13	0.76	0.09	109.83
H3_S	30.59	121.38	0.50	19.70	19.91	5.89	8.02	0.44	0.99	0.11	0.36	0.09	103.94
H4_S	30.44	121.39	0.50	23.50	13.46	6.07	8.00	0.86	0.13	0.15	0.65	0.10	147.87
H5_S	30.64	121.49	0.50	19.70	20.38	5.12	8.03	0.47	0.50	0.12	0.28	0.06	136.70
H6_S	30.52	121.49	0.50	21.40	17.72	6.55	7.94	0.84	0.17	0.18	0.94	0.09	131.27
H7_S	30.38	121.49	0.50	20.30	18.55	6.60	7.99	0.65	0.06	0.17	0.39	0.07	125.42
H8_S	30.31	121.58	0.50	20.10	24.63	6.82	8.03	0.50	0.34	0.12	0.84	0.08	101.28
H9_S	30.41	121.59	0.50	21.10	20.19	6.69	8.00	0.83	0.26	0.15	0.91	0.07	110.95
H10_S	30.54	121.61	0.50	20.70	20.85	6.62	8.03	0.86	0.42	0.12	0.76	0.10	103.38
H11_S	30.67	121.62	0.50	19.80	20.57	5.03	8.03	0.58	0.47	0.13	0.23	0.05	122.25
H12_S	30.69	121.72	0.50	19.80	21.04	5.07	8.02	0.49	0.35	0.14	0.18	0.09	107.70
H13_S	30.53	121.69	0.50	20.00	21.32	6.00	8.01	0.47	0.62	0.11	1.20	0.05	107.77
H14_S	30.54	121.72	0.50	19.50	22.83	6.64	8.02	0.46	0.76	0.12	0.84	0.09	131.81
H15_S	30.40	121.70	0.50	19.20	22.08	7.09	8.04	0.31	0.48	0.13	0.89	0.09	137.70
H16_S	30.29	121.69	0.50	18.30	30.00	7.33	8.03	0.26	0.19	0.16	1.78	0.15	118.28
H17_S	30.29	121.76	0.50	18.00	31.51	5.03	7.98	0.49	0.15	0.13	1.49	0.13	97.42
H18_S	30.38	121.80	0.50	18.20	31.61	5.83	8.00	0.54	0.60	0.12	1.52	0.38	90.45
H19_S	30.51	121.81	0.50	21.30	25.38	6.50	8.04	0.81	0.02	0.16	0.81	0.07	123.81
H20_S	30.58	121.82	0.50	19.90	22.93	6.77	7.99	1.75	0.20	0.15	2.70	0.13	134.40
H1_B	30.45	121.29	7.50	21.30	14.98	5.78	7.97	0.73	1.30	0.08	0.52	0.12	182.13
H2_B	30.58	121.29	13.00	20.10	17.83	6.35	7.99	0.49	0.87	0.07	0.23	0.12	117.82
H3_B	30.59	121.38	13.00	19.80	19.81	4.90	8.01	0.53	1.72	0.09	1.73	0.40	85.59
H4_B	30.44	121.39	7.00	21.20	17.17	6.35	7.97	1.03	1.97	0.10	0.47	0.09	157.01
H5_B	30.64	121.49	11.50	19.90	21.04	4.95	8.02	0.58	2.07	0.10	0.21	0.06	65.95
H6_B	30.52	121.49	7.50	20.30	18.70	6.50	7.98	0.78	1.99	0.10	0.63	0.09	102.87
H7_B	30.38	121.49	9.00	19.80	19.72	6.54	7.97	0.80	5.99	0.11	0.84	0.41	145.07
H8_B	30.31	121.58	9.00	18.80	27.46	6.99	7.96	0.45	3.15	0.10	0.28	0.14	81.32
H9_B	30.41	121.59	10.00	19.60	22.74	6.82	7.96	0.66	8.07	0.11	0.55	0.15	116.14
H10_B	30.54	121.61	8.00	20.10	20.38	6.69	8.01	0.78	2.18	0.10	1.60	0.12	100.66
H11_B	30.67	121.62	9.00	19.60	20.66	4.90	8.01	0.52	3.99	0.11	0.15	0.10	74.97
H12_B	30.69	121.72	9.00	20.10	21.89	6.76	8.01	0.56	0.59	0.05	0.05	0.08	61.62
H13_B	30.53	121.69	8.00	20.20	21.14	6.06	8.02	0.68	1.08	0.08	0.97	0.09	86.26
H14_B	30.54	121.72	10.00	19.20	22.93	6.43	8.02	0.48	0.63	0.05	0.28	0.13	85.09
H15_B	30.40	121.70	8.00	18.90	21.89	7.05	8.01	0.30	5.87	0.11	1.20	0.14	86.15
H16_B	30.29	121.69	12.00	18.00	31.51	7.36	8.00	0.27	1.92	0.10	1.36	0.26	64.99
H17_B	30.29	121.76	13.00	17.80	31.61	5.27	8.00	0.29	2.72	0.10	1.75	0.15	61.47
H18_B	30.38	121.80	14.50	18.30	31.23	5.29	8.01	0.59	1.92	0.10	1.33	0.09	62.13
H19_B	30.51	121.81	9.00	19.00	24.34	5.42	8.02	0.84	2.38	0.10	4.51	0.32	111.49
H20_B	30.58	121.82	8.00	19.30	23.12	6.71	8.02	0.71	2.30	0.10	1.96	0.10	118.93

Note: _S or _B following the site number listed in the sampling ID column denotes surface or bottom water layer.

Supplementary Table S2 Mean (±SD) quantitative PCR measurements for nitrifiers (AOA *amoA* and AOB *amoA* genes), denitrifiers (*nirK* and *nirS* genes) and anammox bacteria (AMB 16S rRNA gene) expressed per litre of sea water. S = Surface water; B = Bottom water

Sample	Gene abundance (copies/L)									
ID	AOA *amoA*	SD	AOB *amoA*	SD	*nirK*	SD	*nirS*	SD	AMB 16S	SD
H1_S	7.41E+05	1.04E+05	2.93E+06	7.16E+05	2.74E+05	8.59E+04	1.24E+07	4.15E+05	3.69E+04	4.35E+02
H2_S	3.16E+06	2.28E+04	1.42E+07	3.92E+05	1.31E+06	2.74E+05	2.53E+07	1.82E+06	3.65E+05	3.72E+04
H3_S	4.27E+07	4.38E+05	1.31E+08	6.87E+06	1.36E+07	2.41E+06	1.61E+08	1.63E+07	2.72E+06	2.90E+05
H4_S	7.52E+05	1.88E+05	4.46E+05	1.53E+06	5.28E+05	8.63E+04	1.69E+07	1.35E+05	2.37E+05	2.45E+04
H5_S	7.98E+06	7.38E+04	4.45E+07	1.58E+05	3.94E+06	3.96E+05	4.15E+07	2.49E+06	6.39E+05	8.85E+04
H6_S	3.00E+06	1.61E+05	1.24E+07	1.08E+06	8.08E+05	9.70E+04	1.83E+07	7.77E+05	1.97E+05	2.77E+04
H7_S	5.81E+05	1.07E+05	1.51E+06	1.87E+05	2.39E+05	1.08E+04	4.07E+06	4.26E+05	5.74E+04	4.24E+03
H8_S	6.25E+06	1.01E+06	2.23E+07	3.76E+06	1.49E+06	1.32E+05	3.52E+07	2.36E+06	5.20E+05	1.33E+05
H9_S	1.39E+06	9.85E+04	6.92E+06	1.65E+06	4.72E+05	9.92E+04	1.25E+07	2.86E+06	1.48E+05	3.62E+04
H10_S	8.86E+06	1.51E+06	4.04E+07	9.74E+06	1.82E+06	3.44E+05	2.34E+07	5.24E+06	6.72E+05	1.76E+05
H11_S	9.48E+06	1.24E+06	5.20E+07	7.00E+05	5.29E+06	6.05E+05	5.29E+07	1.32E+06	8.51E+05	1.24E+05
H12_S	8.83E+06	8.76E+05	4.05E+07	6.10E+05	5.00E+06	7.38E+05	4.06E+07	2.15E+06	5.62E+05	4.76E+04
H13_S	2.95E+06	4.93E+05	1.24E+07	4.23E+06	7.59E+05	2.31E+05	5.79E+07	4.38E+06	3.62E+05	6.27E+04
H14_S	4.07E+06	7.84E+04	2.91E+07	6.42E+05	9.96E+05	4.29E+04	4.42E+07	7.58E+06	4.10E+05	3.80E+04
H15_S	8.40E+06	4.66E+05	3.98E+07	4.28E+05	2.04E+06	5.35E+04	7.96E+07	1.89E+07	8.14E+05	9.45E+04
H16_S	5.60E+06	3.66E+05	2.26E+07	1.51E+06	1.53E+06	1.89E+05	2.89E+07	6.72E+06	2.79E+05	5.83E+04
H17_S	4.38E+06	8.28E+04	1.93E+07	1.05E+06	1.32E+06	6.53E+04	1.99E+07	4.47E+06	2.42E+05	3.24E+04
H18_S	2.42E+06	2.33E+05	8.94E+06	5.03E+05	3.39E+05	3.00E+04	8.16E+06	1.35E+06	1.16E+05	3.25E+04
H19_S	3.06E+05	7.40E+04	7.15E+05	5.37E+04	6.15E+05	1.06E+05	6.86E+06	9.09E+05	3.29E+04	4.26E+03
H20_S	8.82E+06	9.21E+05	4.33E+07	2.90E+06	2.30E+06	3.87E+05	9.80E+07	1.33E+06	6.24E+05	5.03E+04
H1_B	9.23E+06	8.66E+05	2.88E+07	3.37E+06	5.62E+06	1.11E+06	1.03E+08	7.06E+06	6.38E+05	1.66E+05
H2_B	5.38E+06	2.44E+05	4.57E+07	5.29E+06	4.43E+06	5.80E+05	5.46E+07	4.59E+06	7.63E+05	1.08E+05
H3_B	4.28E+06	3.62E+05	1.56E+07	7.39E+05	1.59E+06	1.69E+05	2.04E+07	4.78E+06	2.54E+05	6.15E+04
H4_B	1.21E+07	3.34E+05	1.49E+07	2.05E+07	3.90E+06	7.52E+05	1.24E+08	8.13E+06	7.47E+05	1.85E+05
H5_B	2.00E+07	6.91E+05	1.13E+08	6.17E+06	1.24E+07	1.23E+06	1.25E+08	1.06E+07	1.95E+06	3.33E+05
H6_B	8.01E+06	7.61E+05	8.76E+06	1.96E+07	4.01E+06	1.00E+06	1.07E+08	9.99E+06	3.53E+05	5.35E+04
H7_B	7.43E+07	7.72E+05	4.22E+08	4.55E+07	4.95E+07	8.02E+06	5.90E+08	1.12E+07	9.81E+06	8.77E+05
H8_B	1.53E+07	1.23E+05	8.10E+07	1.33E+07	1.04E+07	1.70E+06	1.16E+08	1.34E+07	1.80E+06	2.91E+05
H9_B	2.20E+07	7.16E+05	1.01E+08	2.19E+07	1.18E+07	1.76E+06	1.43E+08	1.23E+06	2.24E+06	2.73E+05
H10_B	8.04E+06	2.21E+05	4.50E+07	1.23E+07	5.67E+06	1.32E+06	3.92E+07	4.66E+06	4.40E+05	1.68E+05
H11_B	1.74E+05	2.51E+04	3.01E+08	7.92E+06	2.37E+07	5.89E+06	3.02E+08	1.85E+07	4.33E+06	3.09E+05
H12_B	8.79E+05	5.85E+05	6.44E+07	6.09E+05	7.94E+06	2.24E+06	9.90E+07	6.00E+06	1.14E+06	1.54E+05
H13_B	4.44E+06	1.90E+05	2.60E+07	7.07E+06	2.41E+06	6.44E+05	1.92E+07	4.81E+06	5.05E+05	1.21E+05
H14_B	1.92E+07	6.07E+05	7.27E+07	1.79E+07	8.54E+06	2.32E+06	1.76E+08	2.13E+07	2.57E+06	3.79E+04
H15_B	2.01E+07	9.45E+05	1.57E+08	8.34E+06	1.34E+07	1.53E+06	1.49E+08	1.60E+07	3.61E+06	5.64E+05
H16_B	2.04E+06	9.94E+04	1.11E+07	1.10E+06	9.74E+05	2.49E+05	1.32E+07	2.91E+06	2.05E+05	5.93E+04
H17_B	1.97E+07	2.87E+05	1.35E+08	1.50E+07	1.36E+07	1.79E+06	1.37E+08	2.84E+07	2.49E+06	1.93E+05
H18_B	4.53E+07	4.77E+05	2.61E+08	1.14E+07	3.06E+07	1.81E+06	2.58E+08	5.11E+07	5.36E+06	1.09E+05
H19_B	2.48E+07	3.71E+05	1.76E+08	3.08E+07	1.69E+07	1.48E+06	1.70E+08	4.01E+07	3.05E+06	3.94E+05
H20_B	1.89E+07	2.84E+05	1.48E+08	1.15E+07	1.35E+07	1.61E+06	2.30E+08	1.07E+07	2.86E+06	3.96E+05

Supplementary Table S3 Bacterial richness and diversity estimates

Sample ID	Read		97% similarity		
	**Raw**	**Effective**	**OTUs**	**Chao 1**	**Shannon**
H1_S	64002	55571	3280	4505	5.62
H3_S	48089	39936	4376	5700	7.08
H5_S	54975	50047	4547	5629	7.03
H6_S	45772	41978	3781	4942	6.01
H7_S	67694	62719	3288	4458	5.07
H8_S	40247	39117	4022	5043	6.71
H9_S	44496	43516	4016	4897	6.51
H10_S	43570	41596	4175	4990	7.08
H12_S	56459	53741	4445	5407	6.71
H14_S	46636	45013	4276	5211	6.90
H15_S	62959	59963	4713	5560	6.98
H17_S	74278	69053	4559	5484	6.70
H18_S	45457	43838	3326	3805	6.61
H19_S	42243	40844	2076	2930	4.83
H20_S	52085	49147	4273	5287	6.61
H1_B	41901	40069	4300	5262	7.21
H3_B	36107	34117	3744	4563	7.18
H5_B	46192	40656	4356	5483	7.13
H6_B	37511	35727	3963	4838	7.22
H7_B	66351	62524	4817	5650	7.12
H8_B	45363	43982	4211	4969	7.26
H9_B	46179	44378	4471	5409	7.17
H10_B	40729	39079	4322	5457	7.11
H12_B	43596	42571	4288	5304	7.00
H14_B	41604	39482	4305	5377	7.04
H15_B	43812	41422	4510	5456	7.25
H17_B	47429	44005	4465	5337	7.33
H18_B	45626	42879	4439	5323	7.22
H19_B	42968	39642	4415	5453	7.24
H20_B	45438	43403	4470	5519	7.17

OTUs = operational taxonomic units; Chao 1 = Chao’s abundance-based estimator; Shannon = Shannon-Weiner Index.

Supplementary Table S4 Spearman's correlation coefficients (*rho*) between environmental parameters and the abundance of nitrifying genera

	**Variable**	**SPM**	**NH_4^+**	**NO_2^-**	**NO_3^-**	**AOB *amoA***	***N***
Betaproteobacteria	***Nitrosomonas***	**-0.831****	-0.156	**-0.595****	**0.442***	**-0.608****	30
	Nitrosospira	0.138	0.098	0.004	-0.194	-0.182	30
Gammaproteobacteria	***Nitrosococcus***	**0.422***	-0.121	0.289	-0.314	0.338	30

Data in bold indicate significant correlations, $^{*}P$<0.05, $^{**}P$<0.01.

Supplementary Table S5 Spearman's correlation coefficients (*rho*) between SPM and the abundance of denitrifying genera

	Variable	SPM	*N*
Alphaproteobacteria	***Bradyrhizobium***	**0.741****	30
	Paracoccus	-0.172	30
	Hyphomicrobium	0.238	30
	Azospirillum	0.411*	30
	Rhodobacter	**0.489****	30
	Rhizobium	**0.545****	30
	Methylobacterium	**0.495****	30
Betaproteobacteria	***Comamonas***	**0.567****	30
	Thauera	**0.592****	30
	Burkholderia	0.457*	30
	Azospira	0.200	30
	Thiobacillus	-0.261	30
	Ralstonia	0.270	30
	Cupriavidus	0.298	30
	Acidovorax	0.326	30
	Neisseria	0.371*	30
	Alcaligenes	0.455*	30
	Achromobacter	-0.099	30
	Denitratisoma	0.429*	30
Gammaproteobacteria	*Pseudomonas*	0.290	30
	Stenotrophomonas	**0.596****	30
	Pseudoalteromonas	0.430*	30
	Acinetobacter	**0.471****	30
	Halomonas	0.442*	30
	Psychrobacter	-0.322	30
	Marinobacter	-0.101	30
	Alteromonas	0.318	30
	Xanthomonas	0.124	30
Deltaproteobacteria	***Anaeromyxobacter***	**0.519****	30
Epsilonproteobacteria	***Sulfurimonas***	**0.566****	30
	Arcobacter	**0.621****	30
Firmicutes	***Paenibacillus***	**0.477****	30
	Bacillus	0.035	30
	Planomicrobium	0.115	30
	Enterococcus	0.324	30
	Brevibacillus	0.042	30
Bacteroidetes	*Flexibacter*	0.328	30
	Sphingobacterium	**0.502****	30
	Flavobacterium	0.069	30
	Chryseobacterium	**0.492****	30
Actinobacteria	***Arthrobacter***	**0.703****	30
	Streptomyces	0.391*	30
	Nocardia	-0.474**	30
	Micromonospora	0.110	30

Note: Data in bold indicate strongly positive correlations between SPM and the abundance of denitrifying genera (P<0.01).

* P<0.05, ** P<0.01.

Supplementary Table S6 Primer sets used for qPCR

Target gene	Amplicon size (bp)	Primer		Annealing temp (°C)	Reference
		Name	Sequence (5'-3')		
AOA a*moA*	~635	Arch amoA-1F	STAATGGTCTGGCTTAGACG	57	(Abell et al., 2010)
		Arch amoA-2R	GCGGCCATCCATCTGTATGT		
AOB *amoA*	~490	amoA-1F	GGGGTTTCTACTGGTGGT	55	(Abell et al., 2010)
		amoA-2R	CCCCTCKGSAAAGCCTTCTTC		
nirS	425	Cd3aF	GTSAACGTS AAGGARACSGG	57	(Abell et al., 2010)
		R3cd	GASTTCGGRTGSGTCTTGA		
nirK	473	F1aCu	ATCATGGTSCTGCCGCG	58	(Zhang et al., 2014)
		R3Cu	ATCATGGTSCTGCCGCG		
AMB 16S rRNA	280	Brod541F	GAGCACGTAGGTGGGTTTGT	59	(Penton et al., 2006)
		Amx820R	AAAACCCCTCTACTTAGTGCCC		

Supplementary Table S7 16S rRNA-targeted oligonucleotide probes used in this study

**Probe**	**Specificity**	**Sequence (5'-3')**	**Target site[a]**	**FA[b] (%)**	**Reference**
NSO190	Ammonia-oxidizing β-subclass Proteobacteria	CGATCCCCTGCTTTTCTCC	190–208	45	(Mobarry et al., 1996)
DEN67	Methanol-denitrifying cluster	CAAGCACCCGCGCTGCCG	67–86	45	(Lu et al., 2014)
DEN124	Acetate-denitrifying cluster	CGACATGGGCGCGTTCCGAT	124–143	45	(Lu et al., 2014)

[a]16S rRNA position according to Escherichia coli numbering.
[b]FA, formamide concentration in the hybridization buffer.

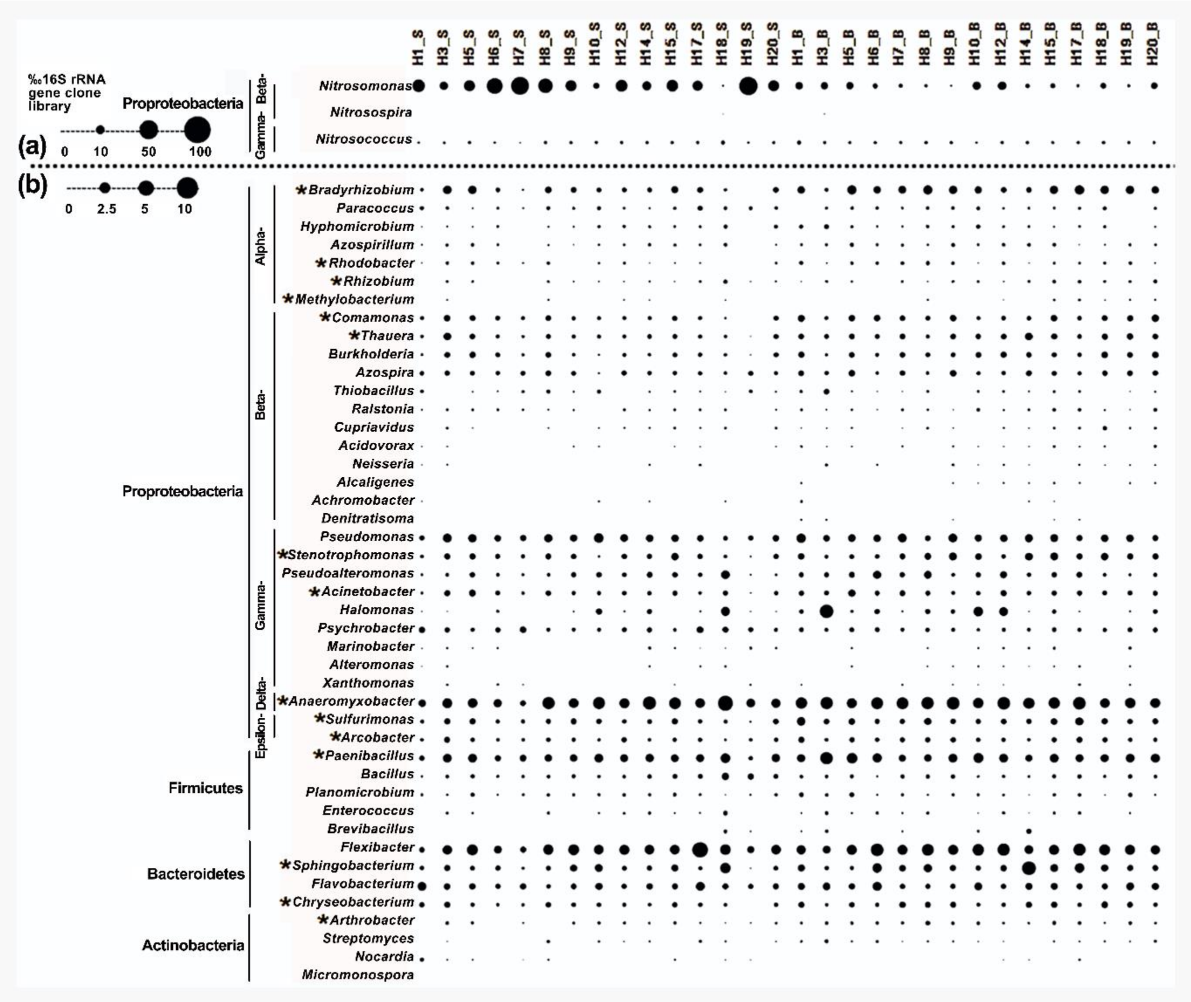

Supplementary Figure S1 Dot plots of relative abundances of (a) nitrifying and (b) denitrifying genes at various sampling sites from the surface (_S) and bottom (_B) of the water column of Hangzhou Bay, revealed by 16S rRNA gene sequencing. Denitrifying genera significantly ($P<0.01$) correlated with SPM are indicated with asterisks, according to the results of Spearman's correlation analyses in Supplementary Table S5.

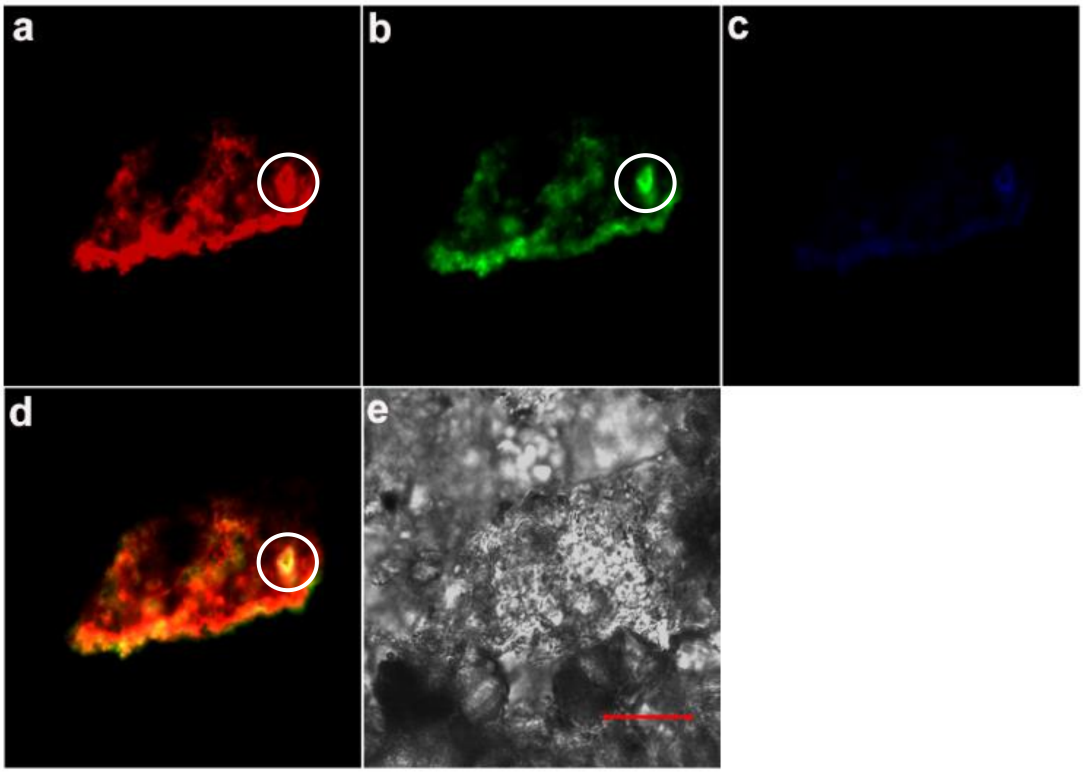

Supplementary Figure S2 Simultaneous *in situ* hybridization of SPM samples in the water column of Hangzhou Bay. Fluorescence micrograph of (a) ammonia-oxidizing bacteria hybridization with Cy3-labeled probe NSO190 (red); (b) acetate-denitrifying cluster hybridization with FAM-labeled probe DEN124 (green); (c) methanol-denitrifying cluster hybridization with Cy5-labeled probe DEN67 (blue); (d) combined image of the three fluorescence micrographs, where the yellow cell aggregates are double labeled with NSO190 and DEN124, and the white cell aggregates are tripled labeled with NSO190, DEN124 and DEN67. A phase contrast-micrograph of the floc section, where the red bar=20 μm, is depicted in (e).

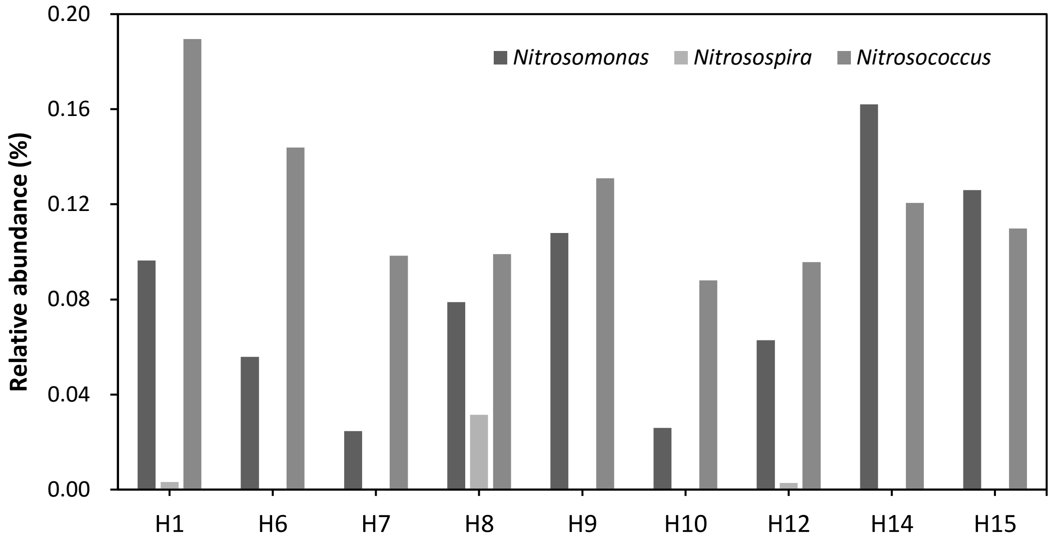

Supplementary Figure S3 Relative abundances of nitrifying genera at sampling sites from Hangzhou Bay sediment, revealed by 16S rRNA gene sequencing.

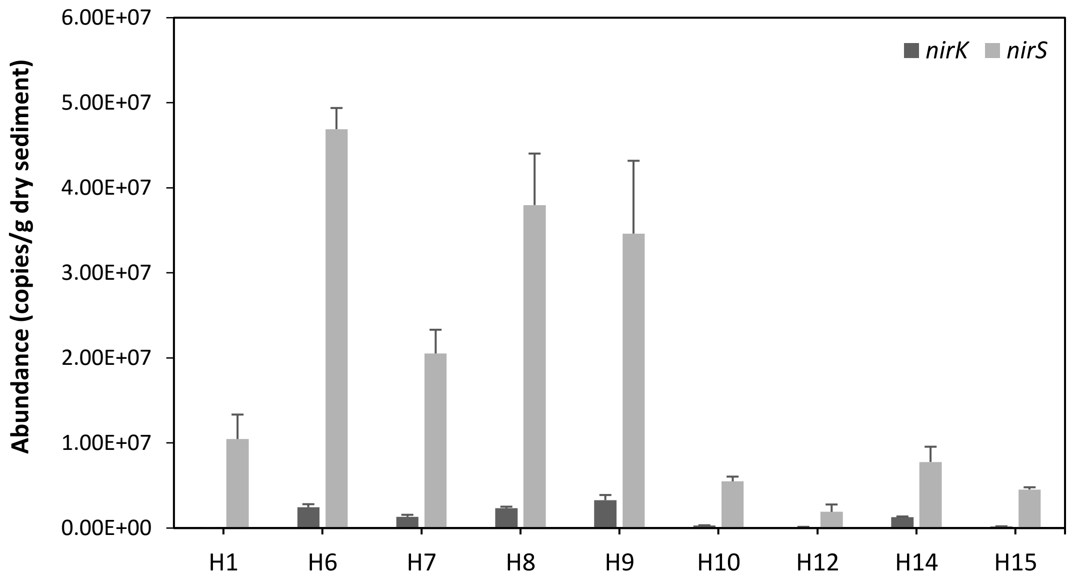

Supplementary Figure S4 Copy numbers (±SD) of *nirK* and *nirS* genes determined for sediment samples (per g dry sediment) of Hangzhou Bay.

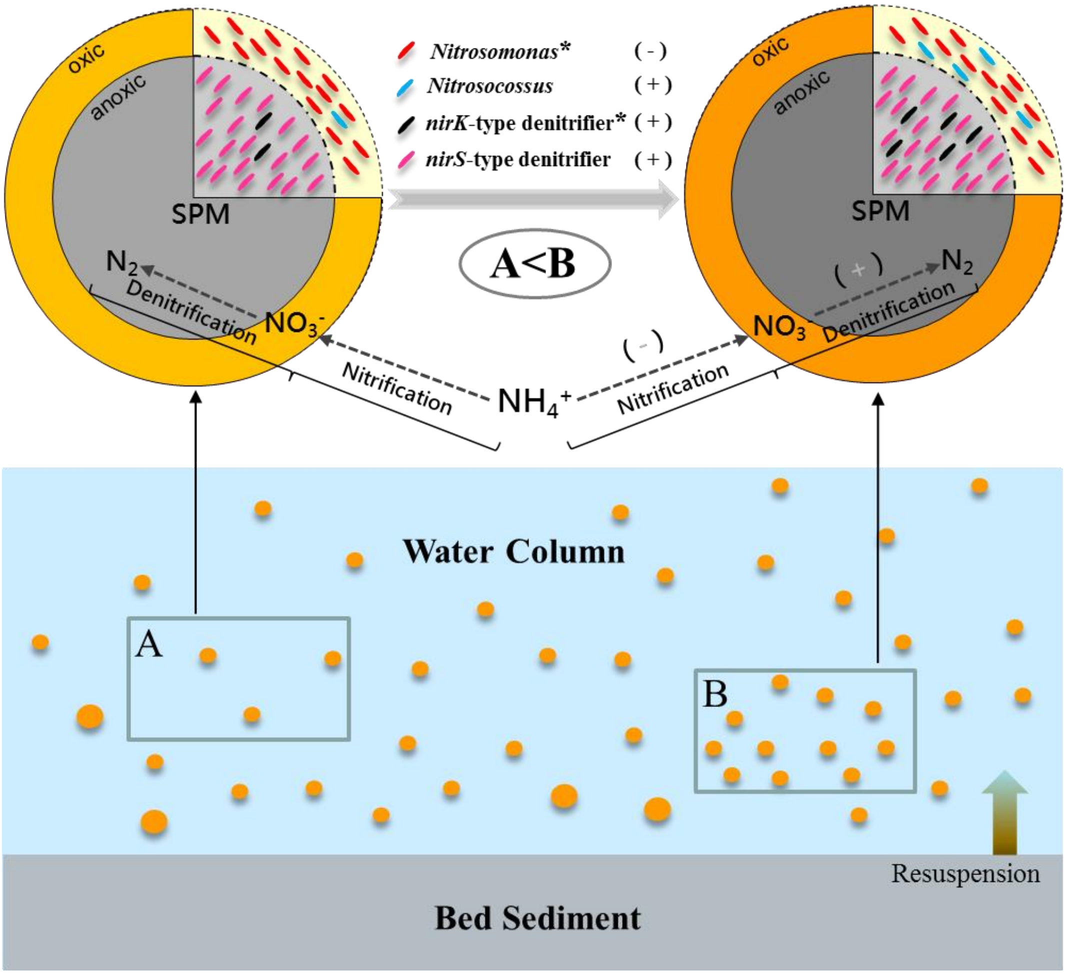

Supplementary Figure S5 Schematic diagram of coupled nitrification-denitrification processes mediated by SPM in the water column of Hangzhou Bay. Asterisk (*) indicates active phylotype in nitrification or denitrification process. The black plus (+) or minus (-) sign represents a significant increase or decrease when SPM concentration increases, while grey plus (+) or minus (-) sign does not indicate a significant relationship.

References

Abell, G.C., Revill, A.T., Smith, C., Bissett, A.P., Volkman, J.K., Robert, S.S., 2010. Archaeal ammonia oxidizers and *nirS*-type denitrifiers dominate sediment nitrifying and denitrifying populations in a subtropical macrotidal estuary. ISME J. 4 (2): 286-300.

Lu, H., Chandran, K., Stensel, D. 2014. Microbial ecology of denitrification in biological wastewater treatment. Water Res. 64 (7): 237-254.

Mobarry, B.K., Wagner, M., Urbain, V., Rittmann, B.E., Stahl, D.A., 1996. Phylogenetic probes for analyzing abundance and spatial organization of nitrifying bacteria. Appl. Environ. Microbiol. 63 (2): 2156-2162.

Penton, C.R., Devol, A.H., Tiedje, J.M., 2006. Molecular evidence for the broad distribution of anaerobic ammonium-oxidizing bacteria in freshwater and marine sediments. Appl. Environ. Microbiol. 72 (10), 6829-6832.

Zhang, X., Agogué, H., Dupuy, C., Gong, J., 2014. Relative abundance of ammonia oxidizers, denitrifiers, and anammox bacteria in sediments of hyper-nutrified estuarine tidal flats and in relation to environmental conditions. CLEAN-Soil Air Water 42 (6): 815-823.

Zhi, E., Song, Y., Duan, L., Yu, H., Peng, J., 2015. Spatial distribution and diversity of microbial community in large-scale constructed wetland of the Liao River Conservation Area. Environ. Earth. Sci. 73 (9):5085-5094.
